# Aggregation-fragmentation and individual dynamics of active clusters

**DOI:** 10.1038/s41467-017-02625-7

**Published:** 2018-02-15

**Authors:** F. Ginot, I. Theurkauff, F. Detcheverry, C. Ybert, C. Cottin-Bizonne

**Affiliations:** Univ. Lyon, Université Claude Bernard Lyon 1, CNRS,UMR 5306, Institut Lumière Matière, F-69622 Villeurbanne, France

## Abstract

A remarkable feature of active matter is the propensity to self-organize. One striking instance of this ability to generate spatial structures is the cluster phase, where clusters broadly distributed in size constantly move and evolve through particle exchange, breaking or merging. Here we propose an exhaustive description of the cluster dynamics in apolar active matter. Exploiting large statistics gathered on thousands of Janus colloids, we measure the aggregation and fragmentation rates and rationalize the resulting cluster size distribution and fluctuations. We also show that the motion of individual clusters is entirely consistent with a model positing random orientation of colloids. Our findings establish a simple, generic model of cluster phase, and pave the way for a thorough understanding of clustering in active matter.

## Abstract (web summary)

The cluster phase of active particles is one instance of the propensity of active matter to self-organize. Combining high-statistics experiments on Janus colloids and simple modeling, Ginot et al. provide a thorough characterization of cluster’s size and motion.

## Introduction

Self-organization, the spontaneous emergence of spatial structures, is a generic phenomenon occurring from atomic to macroscopic length scales, both in inert^1^ and living matter^2^. Even for the restricted class of physical systems at thermodynamic equilibrium, very different behaviors are encountered. The competition between short-range attraction and long-range repulsion driving the microphase separation in block copolymers^3^ or the clustering of proteins and colloids^4^, leads to patterns that are essentially static. However, dynamic self-assembly may also occur with objects that continuously break and form, such as living polymers and wormlike micelles^5^. Systems maintained far from equilibrium by an energy flux are also prone to self-organization, with the emergence of so-called dissipative structures^6^, the instability patterns of continuous media exemplified by the Rayleigh-Bénard convection cells.

The advent of active matter^7,8^ has opened new vista in the already rich landscape of self-organization. Be they micro-tubules bundles^9^, swarming bacteria^2^, birds or fishes^10,11^, active systems usually involve a collection of discrete interacting self-propelled entities. An essential feature is their propensity to exhibit coherent dynamical structures^2,9,12–16^. One prominent instance among those self-organized patterns is the cluster phase that emerges in active particles suspension at low densities, and is arguably its most ‘remarkable’^17^ but ‘mysterious’^16^ property. The competition between self-propulsion and excluded volume is sufficient to induce a self-trapping effect^18–20^, but cluster formation may also involve attractive^21,22^, alignment^23^, phoretic^16,24^ or hydrodynamic^25^ interactions. The dynamics of the cluster phase has multiple facets. Clusters not only exhibit translational and rotational motions, but, in contrast to active systems such as travelling crystals^26^ or colonial choanoflagellates^27^ that retain a permanent structure while moving, they constantly collide, break, and re-form.

In the past decade, bacteria have proven to be a system of choice to uncover the properties of cluster phases. Whereas active crystals^28^ and clusters trapped at the air–liquid interface^25^ have both been reported for rotating bacteria, clustering in rod-shaped bacteria has received the most attention. Experiments with *Bacillus subtilis* involving up to a thousand individuals revealed a wide distribution of cluster size, giant density fluctuations^29^, and highly ordered, scale-invariant clusters^30^. Clustering of myxobacteria exhibits, at a critical cell volume fraction, a size distribution which is scale-free^31^. With the physics of active Brownian rods extensively studied^32,33^, clustering in high density systems with aligning interactions is now well understood. Surprisingly, this is not true for systems at moderate density featuring apolar clustering, where there is no preferred direction in the motion of the clusters. If cluster formation has already been identified^18,34,35^, much has been left unexplored as regards a quantitative understanding. In fact, the size distribution—perhaps the most basic quantity for the cluster phase—has been measured in bacterial systems^21,25,29^ and simulations^19,23,24,36–38^, but so far it has never been reported for the cluster phase of an abiotic system.

Here we close this gap and report a comprehensive characterization of the cluster phase of Janus active particles. We offer a global description of cluster dynamics in a consistent framework. Using systems with thousands of self-phoretic colloids, we track the evolution of hundreds of clusters. We measure the size dependence of fragmentation and aggregation rates, which allows us to rationalize the cluster size distribution and their lifetime. We also analyze the motion of individual clusters, and find that our data is entirely consistent with a parameter-free model assuming random orientation of colloids. Our results identify a simple model of cluster phase and provides a sound basis and methodology to tackle other instances of active clustering and disentangle scenarios of cluster formation.

## Results

### Experimental clusters of Janus microswimmers

**Fig. 1:**
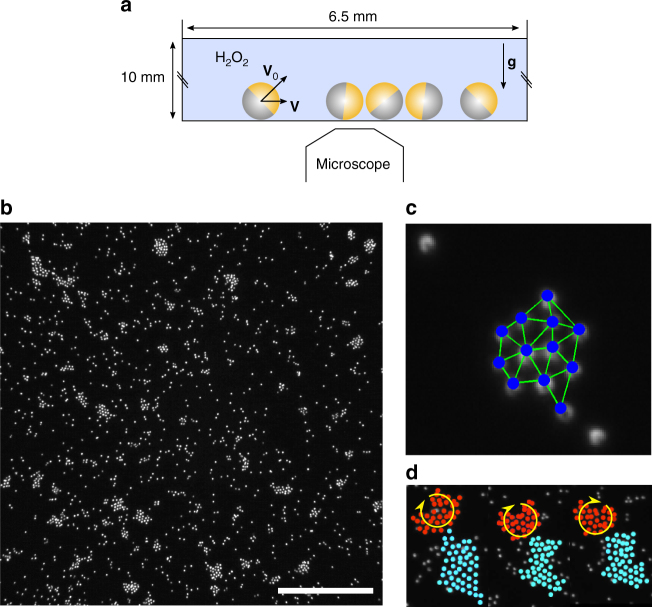
Experimental observation and definition of active clusters. **a** Experimental set-up: sedimented Janus microswimmers immersed in a bath of H_2_O_2_ fuel. **b** Snapshot of the cluster phase, scale bar: 40 μm. **c** Kinetic definition of clusters based on the detection of time persistent triangles of close-packed particles and on the connected component of adjacent triangles (see text and Methods). **d** Series of snapshots (for *t* = 1, 5.4 and 13 s) of cluster-over-cluster rolling motion. A geometric definition of clusters based on a distance-only criterion would identify a single aggregate

The well-controlled experimental set-up, used previously to identify the cluster phase^34^ and investigate sedimentation^39^ and active pressure^40^, is described in the Method section and sketched in Fig. 1a. Briefly, sedimented Janus colloids of micrometric radius *a*_col_ propel themselves by self-diffusiophoresis^41,42^. Their velocity can be chosen in the range *v*_0_ = 1–10 μm s^−1^ and is taken as a measure of activity. The colloid density is set to low values, with an area fraction Φ in the range 5–10%. In those conditions, the system self-organizes in a cluster phase shown in Fig. 1b (Supplementary Movie).

The observation field includes more than 2000 colloids, whose dynamics is individually followed. Short-time motion is well resolved by choosing a camera sampling rate *τ*_s_ = 0.05 s, which is smaller than both the rotational diffusion *τ*_r_ ~ 8 s and the time *a*_col_/*v*_0_ ~ 0.3 s for colloid motion over its own size. The complete evolution of the system is recorded for 250 s, providing large data for analysis. Note that even after a few hours, we detect no sign of the macroscopic phase separation that is expected at higher volume fraction^43^; for all purposes, the system appears in steady state.

Unlike all previous investigations^18,19,22,23,25,36–38,44^, our definition of clusters is not purely geometric, but kinetic. One incentive for the change is the situation depicted in Fig. 1d: two clusters in close vicinity each endowed with their own rotating motion. They would be subsumed in a single cluster with the usual geometric criterion based only on a threshold distance. Other problematic situations include clusters grazing each other or colliding while maintaining their integrity, and single particles wandering at the cluster periphery without actually being incorporated. Our cluster detection algorithm seeks to reproduce the ability of the naked eye to delineate objects. In short (see details in Methods), elementary triangles are introduced on the basis of a Delaunay triangulation and a distance criterion, but only if they fulfill a persistence time *τ*_p_ = 0.5 s. Clusters are then identified as the connected component of elementary triangles sharing one edge (Fig. 1c). Note that as a result, dimers can not exist, an assumption corroborated by direct observation. An immediate benefit of the new definition of clusters is a weak dependence on threshold distance, whereas a purely geometric definition is much more sensitive to this choice. Our clusters are compact, unlike the ramified clusters found previously in simulations^36,37^, and to a very good approximation, they behave as rigid bodies. Their instantaneous motion is therefore entirely characterized by their translational and rotational velocities.

### Aggregation–fragmentation dynamics

**Fig. 2:**
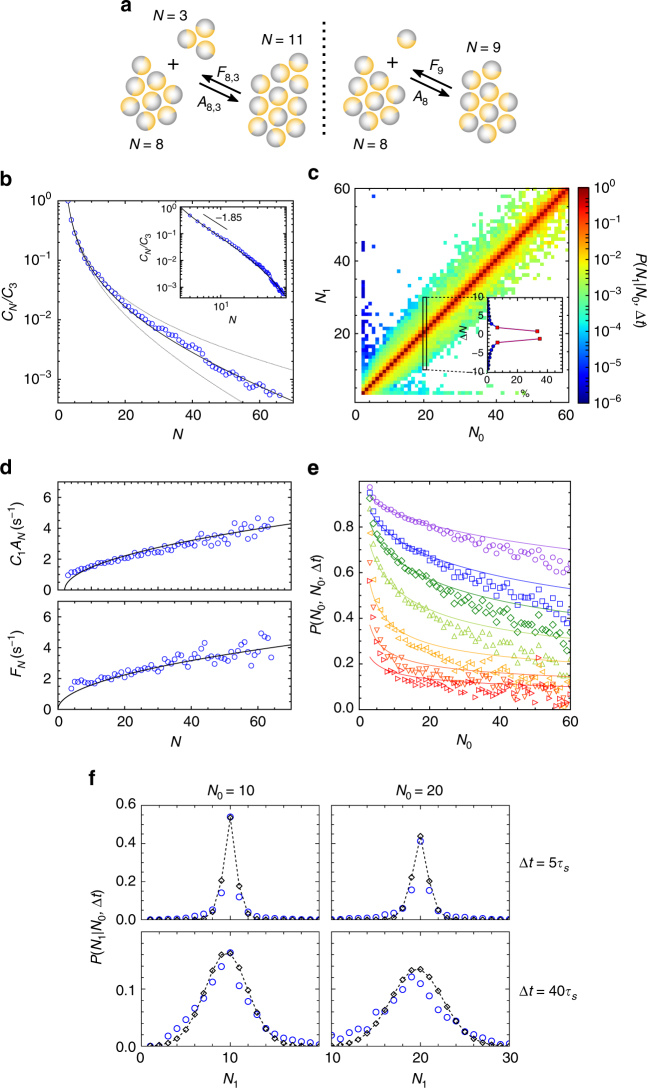
Aggregation–fragmentation process in active clusters. **a** Sketch of the events considered without and with the monomer approximation (left and right respectively).** b–f** Experimental data for activity *v*_0_ = 8.6 μm s^−1^ and area fraction Φ = 9%. **b** Cluster size distribution in semilog scale evidencing the exponential decay at large size. Symbols: experimental data and solid line: theoretical fit (see main text). The dashed lines show the effect of the 2% relative uncertainty on *κ*, obtained upon assuming a 10% relative uncertainty on all data points for CSD and rates. Inset: same data in loglog scale to emphasize the power law regime at small size. **c** Transition matrix showing the probability $P\left( {N_1|N_0,{\mathrm{\Delta }}t} \right)$ for a cluster of size *N*_0_ to have a size *N*_1_ after a time lag Δ*t* = *τ*_s_ = 0.05 s. **d** Aggregation and fragmentation rates for monomeric events. Symbols: experimental data and solid line: theoretical fit (see main text). **e** Probability $P\left( {N_0|N_0,{\mathrm{\Delta }}t} \right)$ that a cluster of size *N*_0_ has the same size after a time Δ*t* = 1, 2, 3, 5, 10, 20 and 40*τ*_s_, from top to bottom. **f** Transition probability $P\left( {N_1|N_0,{\mathrm{\Delta }}t} \right)$ for *N*_0_ = 10 and 20, Δ*t* = 5*τ*_s_ = 0.25 s and 40*τ*_s_ = 2 s. Blue circle: experimental data and black losange: prediction of Eq. (7)

We first focus on the most basic feature of a cluster phase, the Cluster Size Distribution (CSD) *C*_*N*_, a typical result of which is shown in Fig. 2b. Though our definition of clusters is different, we find as in previous works^21,25,36–38,44^, that a power law combined with an exponential cut-off $C_N = N^{ - \gamma }{\mathrm{exp}}\left( { - N{\mathrm{/}}N_{\mathrm{c}}} \right)$ gives a satisfactory description of data, and yields an exponent *γ* = 1.85 ± 0.15 (Fig. 2b, inset). We note however that the range of the power law regime is too limited for a clear-cut evidence, and that the *γ* exponent has not received a clear interpretation. Data also indicate that at large cluster sizes, above *N*_*c*_ = 35, the CSD approaches an exponential decay, a feature less studied so far.

We now seek a microscopic understanding of the CSD, relying on aggregation–fragmentation kinetic models. This framework has a long history dating back to Smoluchowski and a range of applications from polymer chemistry to Saturn’s rings^45,46^. Though more general formulations are possible, we restrict from the outset to aggregation or fragmentation events that are binary:

1 $$[N] + [M]\begin{array}{*{20}{c}} {A_{N,M}C_NC_M} \\ \rightleftharpoons \\ {F_{N,M}C_{N + M}} \end{array}[N + M].$$

As illustrated in Fig. 2a (left), two clusters of size *N* and *M* merge with rate *A*_*N*,*M*_*C*_*N*_*C*_*M*_, while the reverse fragmentation process occurs with rate *F*_*N*,*M*_*C*_*N*+*M*_. At this stage, the complexity of the problem is apparent. On one hand, obtaining the steady-state distribution from the full hierarchy of kinetic equations is a formidable task^45^. On the other hand, if *N*_max_ is the maximal cluster size, the number of rates that needs to be determined is on the order of $N_{{\mathrm{max}}}^{\mathrm{2}}$.

Considerable simplification occurs by introducing the ‘monomer approximation’: not all binary events are considered, but only those involving a monomer^47^. As illustrated in Fig. 2a (right), clusters then grow or decrease in size through the exchange of individual colloids. Our justification for such an approximation is based on the transition matrix $P\left( {N_1|N_0,\tau _{\mathrm{s}}} \right)$, computed from experimental data and shown in Fig. 2c. The element of coordinates (*N*_0_, *N*_1_) is the probability that a cluster of initial size *N*_0_ has size *N*_1_ after one time step *τ*_s_. Looking at off-diagonal terms, one can see that the kinetics is dominated by ±1 and ±2 events. Remembering that dimers do no exist, doublets additions or subtraction actually correspond to a pair of monomeric events. As a consequence, the monomer events typically contribute to 90% of events, and account for the vast majority of aggregation–fragmentation events. Discarding entirely the non-monomeric processes, the kinetic equations then reduce to

2 $$[N] + [1]\begin{array}{*{20}{c}} {A_NC_1C_N} \\ \rightleftharpoons \\ {F_{N + 1}C_{N + 1}} \end{array}[N + 1],$$

with *A*_*N*_ and *F*_*N*_ the aggregation and fragmentation rates for a cluster of size *N*. The CSD is governed by the master equation

3 $$\dot C_N(t) = A_{N - 1}C_1C_{N - 1} - A_NC_1C_N + F_{N + 1}C_{N + 1} - F_NC_N.$$

Imposing a condition of detailed balance, which requires the equality of forward and backward rates in Eq. (2), *F*_*N*+1_*C*_*N*+1_ = *A*_*N*_*C*_1_*C*_*N*_, yields the exact solution for the steady state

4 $$C_N = \left[ {\mathop {\prod}\limits_{m = 2}^N {\kern 1pt} \frac{{A_{m - 1}}}{{F_m}}} \right]C_1^N,$$

with *C*_1_ fixed by normalization. This simple formula applies whatever the *N*-dependence of *A*_*N*_ and *F*_*N*_.

It remains to determine the rates, the essential inputs of an aggregation–fragmentation kinetic model. Although investigated in two recent simulation studies^37,44^, they were never measured in experiments so far. As regards their dependence in cluster size, simple expectations exist (see refinements in refs. 37, 48). Assuming monomers move ballistically and aggregate whenever they collide, the aggregation rate is proportional to the cross-section. For compact clusters and large size, one thus expects *A*_*N*_ ~ *N*^1/2^. In the simplest picture of fragmentation events, perimeter colloids are treated as independent and leave the cluster when pointing outward, leading again to *F*_*N*_ ~ *N*^1/2^. The aggregation and fragmentation rates as deduced from experimental data are plotted in Fig. 2d, together with the expressions

5 $$C_1A_N = \kappa _{\mathrm{A}}(N - 2)^{1/2},\quad F_N = \kappa _{\mathrm{F}}N^{1/2}.$$

Note that *A*_*N*_ vanishes for *N* = 2, consistent with the fact that dimers do not exist. Combining Eqs. (4) and (5), we obtain the cluster size distribution

6 $$\frac{{C_N}}{{C_3}} = \mathop {\prod}\limits_{m = 4}^N \left[ {\frac{{C_1A_{m - 1}}}{{F_m}}} \right] = \frac{{\kappa ^{N - 3}}}{{\sqrt {N(N - 1)(N - 2){\mathrm{/}}6} }},$$

where *κ* = *κ*_A_/*κ*_F_. A two-parameter fit of the three data sets (*C*_1_*A*_*N*_, *F*_*N*_ and *C*_*N*_) yields *κ*_A_ = 0.50 ± 0.005 s^−1^ and *κ*_F_ = 0.52 ± 0.005 s^−1^. The overall satisfactory agreement visible in Fig. 2b, d calls for two comments. First, the $\sqrt N$ dependence of rates is consistent with data, providing support for the basic picture of monomer arrivals and departures, although data range is arguably too limited for a critical test of power law exponent. Second, Eqs. (5) and (6) do capture the small size behavior, which is not a genuine power law, and the tail, which is asymptotically exponential. Note that for completeness, Fig. 2b also displays the envelope associated with relative uncertainties on *κ*. Due to the exponential dependence, significant statistical spread remains despite very small 2% uncertainties, but it has to be appreciated in view of other predictions obtained from the rates alone as we show now.

The CSD is an important feature of the cluster phase, but as a global steady quantity, it tells nothing about how the size of individual clusters fluctuates in time. We get insight on this matter by coming back to the transition matrix $P\left( {N_1|N_0,{\mathrm{\Delta }}t} \right)$: four ‘cross-sections’ are illustrated in Fig. 2f for two cluster sizes and two time intervals. We propose a simple theoretical expectation for the transition matrix by assuming that the arrivals and departures of colloids are two independent Poisson processes, with rate *C*_1_*A*_*N*_ and *F*_*N*_, respectively. If we neglect the size dependence of rate (valid for $N_1 \simeq N_0$), the change in cluster size is the difference between two Poisson variables and thus obeys a Skellam distribution^49^,

7 $$P\left( {N_1|N_0,{\mathrm{\Delta }}t} \right) = \mathrm{e}^{ - (\lambda + \mu )}\left( {\frac{\lambda }{\mu }} \right)^{\frac{{{\mathrm{\Delta }}N}}{2}}I_{\left| {{\mathrm{\Delta }}N} \right|}\left[ {2\sqrt {\lambda \mu } } \right],$$

with *λ* = *C*_1_*A*_*N*_Δ*t*, *μ* = *F*_*N*_Δ*t*, Δ*N* = *N*_1_ − *N*_0_, and *I*_*m*_ the modified Bessel function of order *m*. While this expression does not apply for the smallest sizes and neglects the non-monomeric events that may affect the largest clusters, it gives a very decent description of data for intermediate sizes, both at short and long time (Δ*t* = 0.25 and 2 s) and without any free parameter (Fig. 2f). A complementary view is provided by looking at the probability that the cluster size is the same after a time lapse Δ*t*. The experimental data $P\left( {N_0|N_0,{\mathrm{\Delta }}t} \right)$ is plotted in Fig. 2e for a range of time intervals, and Eq. (7) again captures the main trends. Taken together, our results provide a simple framework which allows to describe in a consistent manner both the microscopic measurement of rates and the resulting CSD and lifetime of active clusters.

### Translational and rotational motions of individual clusters

**Fig. 3:**
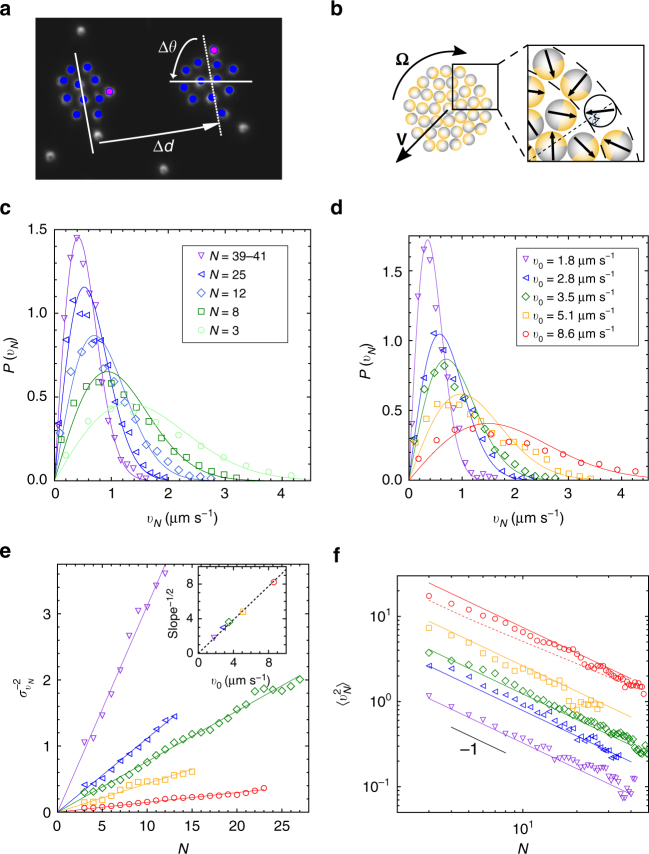
Translational dynamics of individual clusters. **a** Two overlaid snapshots of a cluster showing its translational and rotational motions. The time interval is 10.15 s. **b** Sketch of the cluster model relating the colloids orientation to the global cluster velocities **v** and **Ω**: random orientation model (*α* = *π*) and perimeter model (*α* < *π*). **c**, **d** PDF for the translational velocity modulus *v*_*N*_ of a cluster of size *N*, for fixed activity *v*_0_ = 3.1 μm s^−1^ and various cluster sizes, and for a fixed cluster size (*N* = 12) and different activities. Symbols: experimental data and solid lines: theoretical fits according to Eq. (8), yielding $\sigma _{v_N}$. **e**, **f** Variance and mean square of translational velocity as a function of size and activity (inset). Symbols: experimental data for different activities (same as **d**); solid line: random orientation model Eq. (8); dashed line: perimeter model for the highest activity *v*_0_ = 8 μm s^−1^

**Fig. 4:**
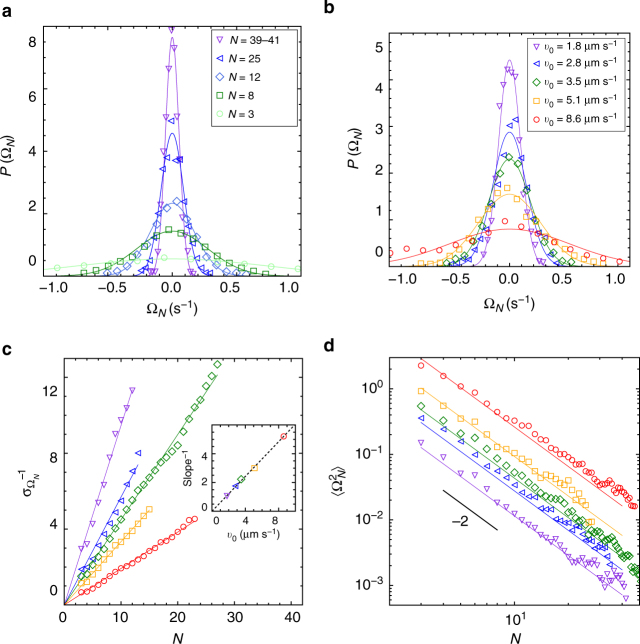
Rotational dynamics of individual clusters. **a**, **b** PDF for the rotational velocity Ω_*N*_ of a cluster of size *N*, for fixed activity *v*_0_ = 3.1 μm s^−1^ and various cluster sizes, and for a fixed cluster size (*N* = 12) and different activities. Symbols: experimental data and solid lines: theoretical fits according to a Gaussian, yielding $\sigma _{{\mathrm{\Omega }}_N}$. **c**, **d** Variance and mean square of rotational velocities as a function of size and activity (inset). Symbols: experimental data for different activities and solid line: random orientation model Eq. (9)

Beyond their evolution in size, the clusters also exhibit a rich dynamics resulting from the underlying activity of constituent particles. At any time, each cluster has instantaneous translational and rotational velocities ***v*** and **Ω** (Fig. 3a, b and Methods). We report in Figs. 3c–f and 4a–d an exhaustive characterization, both as a function of cluster size (*N* = 3, 8, 12, 25, 39–41) and colloid activity (*v*_0_ = 1.8, 2.8, 3.5, 5.1, 8.6 μm s^−1^). We provide not only the mean-squared velocities $\left\langle {v^2} \right\rangle$ and $\left\langle {{\mathrm{\Omega }}^2} \right\rangle$ but exploiting the large statistics gathered, we are in a position to obtain the full probability density functions (PDFs). For all experimental conditions, those PDFs turn out to be very well described by a Gaussian (Figs. 3c, d and 4a, b). Accordingly, they can be entirely characterized by their variance, as obtained from Gaussian fit. As visible in Figs. 3e, f and 4c, d, clear scaling in cluster size emerge for both the translational and rotational velocities, with, respectively, a *N*^−1^ and *N*^−2^ dependence for the variance.

To understand the cluster motions, we now introduce a simple model that extends previous arguments^21,25,28^ and involves only a minimal set of three assumptions. First, the propulsive drive and viscous resistance opposing motion are the same for all colloids, whether isolated or part of a cluster. Second, the clusters behave as rigid bodies, an assumption consistent with their experimental definition. Third, the colloid orientations are random and isotropically distributed.

This random orientation model is easily tractable, since the propulsion force *f*_*N*_ that drives a cluster of size *N* is a sum of independent variables, which for sufficiently large size, is Gaussian. If we call *f*_0_ the force that an active colloid with spontaneous velocity *v*_0_ can exert on a fixed obstacle, and remember that while the swimmer orientation is three-dimensional, only the projection in the plane of motion actually contributes to the dynamics, we find the variance $\left\langle {f_N^2} \right\rangle = (2{\mathrm{/}}3)Nf_0^2$. On the other hand, the cluster friction is simply *ξ*_*N*_ = *Nξ*_0_, where *ξ*_0_ = *f*_0_/*v*_0_ is the friction coefficient of a single colloid. The resulting PDF of translational velocities for a cluster of size *N* (see Supplementary Note 1 for full derivation) is then

8 $$P\left( {v_N} \right) = \frac{{2v_N}}{{\sigma _{v_N}^2}}{\mathrm{exp}}\left[ { - \frac{{v_N^2}}{{\sigma _{v_N}^2}}} \right],\quad \sigma _{v_N}^2 = \left\langle {v_N^2} \right\rangle = \frac{{v_0^2}}{N}.$$

The case of rotational motion can be treated along the same line through the distribution of driving torques generated by each colloid, if we assume that the cluster has the shape of a disk (see Supplementary Note 1). The model predicts again a Gaussian-distributed rotational velocities with variance

9 $$\sigma _{{\mathrm{\Omega }}_N}^2 = \left\langle {{\mathrm{\Omega }}_N^2} \right\rangle = \phi \left( {\frac{{v_0}}{{aN}}} \right)^2,$$

with *ϕ* the packing fraction of colloids in clusters and *a* the effective colloid radius—center-to-center distance—inside clusters. In the following, we take $\phi = \pi {\mathrm{/}}(2\sqrt 3 ) \simeq 0.91$ and the measured value *a* = 1.6 μm.

The predictions of the random orientation model agree surprisingly well with our entire set of experimental data. It rationalizes not only the Gaussian shapes of PDFs but as shown in Fig. 3c,d (resp. Fig. 4a,b), provides a quantitative agreement for translational (resp. rotational) velocity distribution. The experimental dependency of the velocity variances on cluster size *N* or particles activity *v*_0_ are fully captured by Eqs. (8) and (9). Remarkably, this perfect matching is obtained without free parameter.

## Discussion

We now examine the resulting insights on the internal mechanisms governing cluster formation and cohesion, and to do so, we reconsider in turn the three assumptions underlying our model of active cluster.

Our data set and analysis support the idea that the motile strength *f*_0_ for individual Janus colloid is the same for particles in a cluster or alone. This is in contrast with bacterial systems, where the crowding may hinder the flagella’s motion or efficiency resulting in a reduced motility within the clusters^21,28^. If not a priori obvious, this environment-independent motility may be a benefit of our experimental design, where a two-dimensional layer of sedimented colloids is immersed in a three-dimensional fuel bath. Such set-up optimally preserves a constant chemical feed irrespective of the surface environment.

Even if a few large clusters are observed to break apart, the overwhelming majority of clusters behave as rigid and cohesive objects. This suggests the presence of an attractive force *f*_a_ between colloids. Our observations put constraints on its strength: if $f_{\mathrm{a}} \ll f_0$, its influence would be negligible, whereas if $f_{\mathrm{a}} \gg f_0$, irreversible aggregation into a macroscopic condensed domain would ensue. This implies that *f*_a_ and *f*_0_ have the same order of magnitude. Now, we note that when the activity is increased five-fold, there is no discernible change of behavior, suggesting that *f*_a_ may increase with activity and that the attraction may be phoretic in nature. This is a new hint of the existence of an intrinsic interaction between Janus swimmers resulting from chemical cloud generated by neighbors, as previously suggested for our system^40^.

Though entirely consistent with the data, the success of the random orientation is surprising at first sight. Indeed, the self-trapping mechanism for clusters formation implies that perimeter colloids point mostly inwards^16^, as suggested by direct observation in another system^18^. To see how those effects should affect the cluster velocities, we propose a refinement of the model. The idea is to split the cluster particles in two sub-populations: a core, which obeys the random orientation assumption and a perimeter, where possible orientations are constrained. For concreteness, the allowed orientations are still uniform but restricted to the range [−*α*, *α*] around the direction of the center (Fig. 3b). For this “perimeter model”, the mean-squared cluster velocity is now (Supplementary Note 2)

10 $$\left\langle {v_N^2} \right\rangle = \frac{{v_0^2}}{N}\left[ {{\mathrm{\Phi }}_{\mathrm{b}} + \frac{{{\mathrm{\Phi }}_{\mathrm{p}}}}{4}(5 + {\mathrm{cos}}{\kern 1pt} \alpha ){\kern 1pt} {\mathrm{sin}}^2\left( {\frac{\alpha }{2}} \right)} \right],$$

where Φ_b_ = *N*_b_/*N* is the fraction of bulk particles in a cluster of size *N*, and Φ_p_ = 1 − Φ_b_. In practice, a simple approximation is ${\mathrm{\Phi }}_{\mathrm{b}} = ( {1 - 2\sqrt {\phi {\mathrm{/}}N} } )^2{\mathrm{\Theta}}(N - 4)$ with *ϕ* the packing fraction defined in Eq. (9) and Θ the Heaviside function. This expression arises from the large cluster limit to which the Heaviside cutoff is incorporated to ensure that the smallest clusters are perimeter-only objects. The case of rotational velocities can be treated in a similar fashion (Supplementary Note 2).

With perimeter colloids pointing inwards and their contribution tending to cancel each other, the perimeter model predicts a reduction in cluster velocities. The effect is most pronounced at low *α* and small size, where the perimeter contribution dominates. To avoid fitting parameter at this semi-quantitative stage, we set *α* to its simplest and most natural value of *π*/2. The resulting prediction for mean square velocities consistently falls below the data (not shown), and compared to the random orientation model, agreement rather deteriorates. The only possible exception is the translational velocity at highest activity *v*_0_ = 8.6 μm s^−1^, where the departure from the random orientation model might be ascribed to perimeter effects (Fig. 3f). As regards the rotational velocities, the predictions of the perimeter model for *α* = *π*/2 coincide with the random orientation model (recovered with *α* = *π*), and no further support can be drawn from Fig. 4d. Overall, this simple perimeter model—which in particular does not account for possible attraction between particles—appears less satisfactory than the random orientation model, which provides the simplest and most consistent description of our data. As a final note, we remark that we applied Occam’s razor again when discarding entirely hydrodynamic interactions^25,38,50^. At no point did the need arise to introduce their effect.

In conclusion, by exploiting high-statistics experiments, we have thoroughly characterized the cluster phase of spherical Janus microswimmers, providing an elementary but complete description of the system. While a near perfect alignment is observed for clusters of active polar matter^29,30^, a simple random orientation model perfectly accounts for the individual dynamics of our clusters. Furthermore, a simple approach for the aggregation and fragmentation mechanisms gives an excellent description of the cluster size distribution and lifetime. Since our modelling involves only straightforward ingredients, we believe it will constitute a sound basis upon which more sophisticated treatments can be built, thus helping to develop a generic framework for the description of active clusters.

Looking forward, two directions emerge for future work. First, regarding the influence of density: no obvious change could be detected within the restricted interval considered here, but it remains to explore a wider range to probe the validity of the monomer assumption and to delineate the cluster phase boundaries. Second, a grand challenge is to disentangle the generic and specific aspects of cluster formation. In particular, we can ask to what extent the cluster properties depend on the type of interactions between particles. A complete answer will require not only a full characterization of the cluster phase as done here but also a detailed view of the propulsion mechanisms and interactions in synthetic active matter^51^. It will be important to shed light on the relations or differences between our living clusters and patterns observed numerically for phoretically active colloids^16^. From recent examples of phoretic and magnetic interactions^16,52^, it is already apparent that a rich phenomenology can be found in the cluster phase, which appears as a promising frontier of active matter.

## Methods

### Experimental set-up

Gold colloids of nominal radius *a*_col_ = 1.1 ± 0.1 μm were synthesized^53^ and half-coated with Platinum to form Janus microswimmers when immersed in hydrogen peroxide (H_2_O_2_) solutions^34,40,41^. Due to their high density $\rho \simeq 11{\kern 1pt}$ g cm^−3^, the colloids immediately sediment onto the flat bottom of the experimental cell to form a bidimensional layer of active particles, whose area fraction Φ is determined experimentally (Fig. 1a). Such a configuration of active Janus particles immersed in a bulk solution loaded with H_2_O_2_ fuel ensures a continuous and constant activity of the microswimmers over periods exceeding half an hour for which stationary-state properties can be extensively investigated. By tuning the H_2_O_2_ concentration *c* in the range [10^−4^, 10^−2^] v/v%, it is possible to vary the self-propulsion velocity of the colloids *v*_0_. In practice, the latter is experimentally determined in each experiment and varies from 1 to 10 μm s^−1^. The area fraction can not be finely controlled but is within the range 5–10%.

### Cluster definition

We define triangles of closest neighbors using Delaunay triangulation and only keep triangles with all sides smaller than 3.4 μm. We also add a time constraint: triangles must respect the spatial condition during *τ*_p_ = 0.5 s to be kept. Triangles are defined as adjacent if they share one edge (two particles). The clusters are computed as the connected components of adjacent triangles (see Fig. 1c).

### Translational and rotational velocities

For a cluster of *N* particles with individual velocities **v**_*i*_ and positions **r**_*i*_ with respect to the cluster center-of-mass, we define the translational velocity **v** as the mean velocity of the particles inside the cluster ${\bf{v}} = \frac{1}{N}\mathop {\sum}\nolimits_{i = 1}^N {\bf{v}}_i$. To obtain the rotational velocities **Ω**, we first compute the angular momentum ${\boldsymbol{\sigma }} = \mathop {\sum}\nolimits_{i = 1}^N {\kern 1pt} {\bf{r}}_i \times {\bf{v}}_i$ and the moment of inertia $I = \mathop {\sum}\nolimits_{i = 1}^N {\kern 1pt} {\bf{r}}_i^2$, and obtain the rotational velocity **Ω** from **σ** = **Ω***I*.

### Data availability

All the relevant data are available from the authors on request.

## Electronic supplementary material

Supplementary Information

Description of Additional Supplementary Files

Supplementary Movie 1
