# Supplementary material for: Aggregation-fragmentation and individual dynamics of active clusters
Source: Nat Commun. 2018 Feb 15;9:696. doi: 10.1038/s41467-017-02625-7 (PMC5814572; doi:10.1038/s41467-017-02625-7)
Supplement: Supplementary file 1 — Supplementary Information [file 41467_2017_2625_MOESM1_ESM.pdf]

Supplementary Note 1

Random orientation model

We provide a detailed derivation of the results quoted for the random orientation and perimeter models.

Notations. If a random variable x is Gaussian with zero mean and variance $\langle x^2\rangle$, we write

$$P(x) = \mathcal{G}_1(x, \langle x^2\rangle) \equiv \frac{1}{\sqrt{2\pi\langle x^2\rangle}} \exp\left[-\frac{x^2}{2\langle x^2\rangle}\right]. \tag{1}$$

Similarly, if a bidimensional vector $\mathbf{v}$ is Gaussian with zero mean and variance $\langle v^2\rangle$, we write

$$P(\mathbf{v}) = \mathcal{G}_2(\mathbf{v}, \langle v^2\rangle) \equiv \frac{1}{\pi\langle v^2\rangle} \exp\left[-\frac{\mathbf{v}^2}{\langle v^2\rangle}\right]. \tag{2}$$

For clarity of notation, here we drop the N-subscript indicating explicitly the size dependence of quantity but it is implied everywhere.

Translational velocities

Though eventually applied for three-dimensional orientations ($d = 3$), the model is first presented with two-dimensional orientations ($d = 2$) for clarity.

d=2. Each colloid exerts an effective propulsive force $\mathbf{f}$ with fixed modulus f_0 and whose orientation is randomly chosen in a uniform distribution. The total propulsive force $\mathbf{F} = \sum_{n=1}^{N} \mathbf{f}_n$ is the sum of identical and independent variables with finite variance. By the central limit theorem, its distribution approaches for large N a Gaussian with zero mean and variance $\langle F^2\rangle$, namely

$$P(\mathbf{F}) = \mathcal{G}_2(\mathbf{F}, \langle F^2\rangle), \qquad \langle F^2\rangle = N{f_0}^2. \tag{3}$$

To obtain the cluster velocity, we use the fact that each colloid of a cluster opposes motion exactly as free colloids (which have velocity v_0 and friction coefficient $\xi_0 = f_0/v_0$). The distribution of cluster velocity $\mathbf{v} = \mathbf{F}/(N\xi_0)$ is then

$$P(\mathbf{v}) \;=\; \mathcal{G}_2(\mathbf{v}, \langle v^2\rangle), \qquad \langle v^2\rangle = \frac{{v_0}^2}{N}. \tag{4}$$

d=3. When three-dimensional, the colloid orientation is assumed to be uniformly distributed on a unit sphere. Because all colloids remain in the same plane, we consider only the projection of the propulsive force in this plane. Denoting by $\mathbf{F}$ the projected force, and retracing the steps above, one finds

$$P(\mathbf{F}) \;=\; \mathcal{G}_2(\mathbf{F}, \langle F^2\rangle), \qquad \langle F^2\rangle = \frac{2}{3}N{f_0}^2. \tag{5}$$

Here the velocity of a free particle is not constant any more, but it can be characterized by its mean quadratic value. We define v_0 as the mean square velocity of a free particle, that is ${v_0}^2 \equiv \langle v^2\rangle_{\text{free}} = 2/3(f_0/\xi_0)^2$. With this definition, Supplementary Equation (4) applies again, and considering the modulus, leads to Equation (8) of the main text.

Rotational velocities

Because of hard core repulsion, correlations exist in colloid positions, hence in their torques too. We circumvent the difficulty by neglecting altogether correlation in positions: each colloid assumes a random orientation and position. Because the individual contributions are now independent and identical variables, the total torque for a large cluster is once again a Gaussian. Since it has zero mean, we only need to compute its second moment.

To do so, let's compute the contribution of a single colloid, treating directly the case of three-dimensional orientations, and considering only the torque in the out-of-plane direction. The joint probability density $P(\mathbf{r}, f_\perp)$ that a colloid falls at position $\mathbf{r}$ from the cluster center and with a propulsive force having a component $f_\perp$ perpendicular to $\mathbf{r}$, is

$$P(\mathbf{r}, f_\perp) = \frac{1}{\pi R^2} \times \frac{1}{2f_0}, \qquad |\mathbf{r}| \leqslant R, \quad |f_\perp| \leqslant f_0. \tag{6}$$

The first term in the RHS assumes that the cluster is a disk of radius R. The second term arises because for vector of modulus f_0 and random isotropic orientation, the component along an arbitrary axis has a flat distribution in the range $[-f_0, f_0]$. The torque for a single colloid $\tau = rf_\perp$ can now be obtained by writing first $P(\mathbf{r}, \tau)$ and then integrating over position, with the result

$$P(\tau) = \frac{1}{\tau_\mathrm{m}}\left(1 - \frac{|\tau|}{\tau_\mathrm{m}}\right), \quad \langle\tau^2\rangle = \frac{\tau_\mathrm{m}^2}{6}, \quad \tau_\mathrm{m} = Rf_0, \tag{7}$$

where τ_m is the maximal value of the torque. Finally, introducing the total torque $\mathcal{T} = \sum_{n=1}^{N} \tau_n$, we have for sufficiently high N,

$$P(\mathcal{T}) = \mathcal{G}_1(\mathcal{T}, \langle\mathcal{T}^2\rangle), \qquad \langle\mathcal{T}^2\rangle = N\frac{R^2 {f_0}^2}{6}. \tag{8}$$

To access the distribution of rotational velocities $P(\Omega)$, it remains to express the resistant torque $\mathcal{T}_R$. Assuming that each colloid in the cluster resists motion as in its free state, one has

$$\mathcal{T}_R \ = \int_0^R \frac{2\pi r \mathrm{d}r\phi}{\pi a^2} \times r \times \Omega r \xi_0 = \frac{N^2 a^2 \xi_0}{2\phi}\Omega, \tag{9}$$

where we used $\phi\pi R^2 = N\pi a^2$. Upon using $\mathcal{T}_R = \mathcal{T}$ and Supplementary Equation (9), the final expression for $P(\Omega)$ is

$$P(\Omega) \ = \ \mathcal{G}_1(\Omega, \langle\Omega^2\rangle), \qquad \langle\Omega^2\rangle = \phi\left(\frac{v_0}{aN}\right)^2. \tag{10}$$

which is Equation (9) of the main text.

Supplementary Note 2

Perimeter model

Translational velocities

d=2. Again we treat first the case of two-dimensional orientations for clarity. In contrast with the N_b colloids in the bulk, the N_p colloids in the perimeter, assumed to form a regular polygon, do not have isotropic orientation. Rather, their orientation deviates from the direction of the center by an angle uniformly distributed in the interval $[-\alpha, \alpha]$. The distribution of perimeter propulsive force $\mathbf{F}_\mathrm{p} = \sum_{n=1}^{N_\mathrm{p}} \mathbf{f}_n$ is difficult to evaluate exactly for arbitrary value of N_p. Instead, we postulate that it can be described by a Gaussian, which requires only to characterize the second moment. Then

$$P(\mathbf{F}_\mathrm{p}) = \mathcal{G}_2(\mathbf{F}_\mathrm{p}, \langle F_\mathrm{p}^2\rangle), \qquad \langle F_\mathrm{p}^2\rangle = N_\mathrm{p}{f_0}^2\left(1 - \mathrm{sinc}^2(\alpha)\right), \tag{11}$$

where $\mathrm{sinc}(x) = \sin(x)/x$ is the cardinal sine.

The calculation of $\langle F_\mathrm{p}^2\rangle$ runs as follows. If $\mathbf{e}_r(\theta)$ is the radial unit vector making an angle θ with respect to a reference axis, one has $\mathbf{f}_n = f_0\,\mathbf{e}_r(\theta = 2\pi(n-1)/N_\mathrm{p} + \varphi_n)$, with φ_n the random deviation angle. Then

$$\langle F_\mathrm{p}^2\rangle \;=\; \langle\sum_{n=1}^{N_\mathrm{p}} \mathbf{f}_n \cdot \sum_{n'=1}^{N_\mathrm{p}} \mathbf{f}_{n'}\rangle = N_\mathrm{p} {f_0}^2 + \sum_{n=1}^{N_\mathrm{p}} \sum_{\substack{n'=1\\ n'\neq n}}^{N_\mathrm{p}} \langle \mathbf{f}_n \cdot \mathbf{f}_{n'}\rangle. \tag{12}$$

Now, for $n' \neq n$, performing the average gives

$$\begin{aligned}\langle \mathbf{f}_n \cdot \mathbf{f}_{n'}\rangle &= {f_0}^2 \langle \cos\left(2\pi(n-n')/N + \varphi_n - \varphi_{n'}\right)\rangle, \\ &= {f_0}^2 \int_{-\alpha}^{\alpha} \frac{\mathrm{d}\varphi_n}{2\alpha} \int_{-\alpha}^{\alpha} \frac{\mathrm{d}\varphi_{n'}}{2\alpha} \cos\left(2\pi(n-n')/N_\mathrm{p} + \varphi_n - \varphi_{n'}\right), \\ &= {f_0}^2 \left(\frac{\sin\alpha}{\alpha}\right)^2 \cos\left(2\pi(n-n')/N_\mathrm{p}\right).\end{aligned} \tag{13}$$

Supplementary Equation (11) is obtained upon using the identity

$$\sum_{n=1}^{N_\mathrm{p}} \sum_{\substack{n'=1\\ n'\neq n}}^{N_\mathrm{p}} \cos\left(2\pi(n-n')/N_\mathrm{p}\right) = -N_\mathrm{p}. \tag{14}$$

Note that as expected, for the particular case $\alpha = \pi$, the perimeter contribution identifies with the bulk one.

The propulsive force $\mathbf{F}_\mathrm{b}$ induced by bulk colloids is given by the random orientation model considered above. As the sum of two Gaussian distributed variables, the total propulsive force $\mathbf{F} = \mathbf{F}_\mathrm{b} + \mathbf{F}_\mathrm{p}$ is itself a Gaussian with

$$P(\mathbf{F}) = \mathcal{G}_2(\mathbf{F}, \langle F^2\rangle), \qquad \langle F^2\rangle = \langle F_\mathrm{b}^2\rangle + \langle F_\mathrm{p}^2\rangle = N{f_0}^2\left[\Phi_\mathrm{b} + \Phi_\mathrm{p}\left(1 - \mathrm{sinc}^2(\alpha)\right)\right]. \tag{15}$$

with $\Phi_\mathrm{b} = N_\mathrm{b}/N$ the fraction of bulk particles in a cluster of size N and $\Phi_\mathrm{p} = 1 - \Phi_\mathrm{b}$. Proceeding as above, the distribution of cluster velocity is

$$P(\mathbf{v}) \;=\; \mathcal{G}_2(\mathbf{v}, \langle v^2\rangle), \qquad \langle v^2\rangle = \frac{{v_0}^2}{N}\left[\Phi_\mathrm{b} + \Phi_\mathrm{p}\left(1 - \mathrm{sinc}^2(\alpha)\right)\right]. \tag{16}$$

d=3. In the case of three-dimensional orientations, the colloid orientation is now assumed to be uniformly distributed on a spherical cap of opening angle α around the center direction. Because all colloids remain in the same plane, we consider only the projection of the propulsive force in this plane. Denoting by $\mathbf{F}_\mathrm{b}$ and $\mathbf{F}_\mathrm{p}$ the projected forces, and retracing the steps above, one finds

$$P(\mathbf{F}_\mathrm{b}) \;=\; \mathcal{G}_2(\mathbf{F}_\mathrm{b}, \langle F_\mathrm{b}^2\rangle), \qquad \langle F_\mathrm{b}^2\rangle = \frac{2}{3} N_\mathrm{b} {f_0}^2, \tag{17}$$

$$P(\mathbf{F}_\mathrm{p}) \;=\; \mathcal{G}_2(\mathbf{F}_\mathrm{p}, \langle F_\mathrm{p}^2\rangle), \qquad \langle F_\mathrm{p}^2\rangle = \frac{2}{3} N_\mathrm{p} {f_0}^2 h_v(\alpha), \quad h_v(\alpha) = \frac{1}{4}(5 + \cos\alpha)\sin^2(\alpha/2). \tag{18}$$

Putting everything together, the final expression for the distribution of cluster velocity $P(\mathbf{v})$ in the perimeter model is

$$P(\mathbf{v}) = \mathcal{G}_2(\mathbf{v}, \langle v^2\rangle), \qquad \langle v^2\rangle = \frac{{v_0}^2}{N}\left[\Phi_\mathrm{b} + \frac{\Phi_\mathrm{p}}{4}(5 + \cos\alpha)\sin^2\left(\frac{\alpha}{2}\right)\right], \tag{19}$$

as given in Equation (10) of the main text

Rotational velocities

As regards the perimeter contribution to torque, each colloid yields the same distribution, which can be computed exactly. We note l the distance between the cluster center and the center of perimeter colloids. Proceeding as above, one finds for the sum $\mathcal{T}_\mathrm{p} = \sum_{n=1}^{N_\mathrm{p}} \tau_n$ the result

$$P(\mathcal{T}_\mathrm{p}) \;=\; \mathcal{G}_1(\mathcal{T}_\mathrm{p}, \langle \mathcal{T}_\mathrm{p}^2\rangle), \qquad \langle \mathcal{T}_\mathrm{p}^2\rangle = N_\mathrm{p} {f_0}^2 l^2 \frac{h_\Omega(\alpha)}{3}, \tag{20}$$

$$l \;=\; \frac{a}{2\sin(\pi/N_\mathrm{p})}, \tag{21}$$

$$h_\Omega(\alpha) \;=\; (2 + \cos\alpha)\sin^2(\alpha/2). \tag{22}$$

The distribution for the total torque $\mathcal{T} = \mathcal{T}_{\rm b} + \mathcal{T}_{\rm p}$ then reads

$$P(\mathcal{T}) \;=\; \mathcal{G}_1(\mathcal{T}, \langle \mathcal{T}^2 \rangle), \qquad \langle \mathcal{T}^2 \rangle = a^2 {f_0}^2 \left[\frac{N_{\rm b}^2}{6\phi} + \frac{N_{\rm p}}{12 \sin^2(\pi/N_{\rm p})} h_\Omega(\alpha) \right]. \tag{23}$$

Now the resistant torque is

$$\mathcal{T}_R \;=\; \int_0^{R_{\rm b}} \frac{2\pi r {\rm d}r\phi}{\pi a^2} \times r \times \Omega r \xi_0 + N_{\rm p} \Omega l^2 \xi_0 = \Omega a^2 \xi_0 \left[\frac{N_{\rm b}^2}{2\phi} + \frac{N_{\rm p}}{4 \sin^2(\pi/N_{\rm p})} \right] = \Gamma \Omega, \tag{24}$$

where we used $\phi R_{\rm b}^2 = N_{\rm b} a^2$. Upon using $\mathcal{T}_R = \mathcal{T}$ and Supplementary Equation (24), the final expression for $P(\Omega)$ is $\Gamma P(\Gamma\Omega)$, where P is given by Supplementary Equation (23).

As shown in Supplementary Figure 1, the influence of the opening angle α on the variance of translational and rotational velocities is quite different. In the range $\alpha \in [\pi/2, \pi]$, which is most relevant here, the function $h_v(\alpha)$ is steadily increasing. In contrast, $h_\Omega(\alpha)$ depends only weakly on α. Indeed, at both ends of the interval, $h_\Omega(\alpha)$ takes the same value ($h_\Omega(\pi/2) = h_\Omega(\pi)) = 1$) and in between, it reaches a maximum at $\alpha = 2\pi/3$ that is only slightly different from unity ($= 9/8$). The difference of behavior between $h_v(\alpha)$ and $h_\Omega(\alpha)$ is most pronounced for $\alpha = \pi/2$.

As a final remark, note that in both the random orientation model and the perimeter model, we consider only the instantaneous velocity of a cluster. To do so does not require to specify a dynamics for the colloids orientation. If such dynamics is postulated, one could extend the model and address the temporal evolution of cluster velocity as characterized, for instance, by the velocity correlation function. In practice, however, aggregation or fragmentation occur so frequently that the cluster size changes before the relaxation in velocity occurs, thus preventing such analysis.

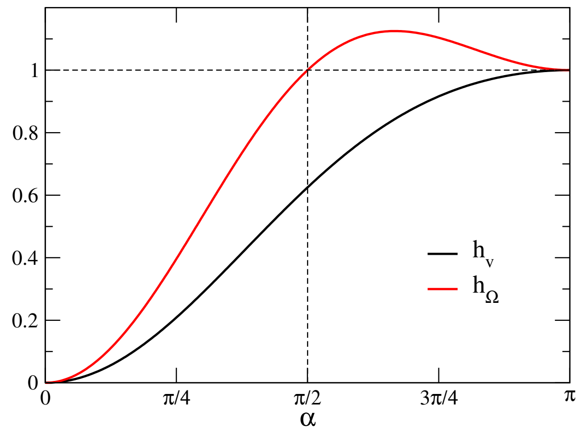

Supplementary Fig. 1: Functions $h_v(\alpha)$ and $h_\Omega(\alpha)$ as defined in Supplementary Equations. (18) and (22).
