# Supplementary material for: Aggregation-fragmentation and individual dynamics of active clusters
Source: Nat Commun. 2018 Feb 15;9:696. doi: 10.1038/s41467-017-02625-7 (PMC5814572; doi:10.1038/s41467-017-02625-7)
Supplement: Supplementary file 2 — Description of Additional Supplementary Files [file 41467_2017_2625_MOESM2_ESM.pdf]

Description of Additional Supplementary Files

File Name: Supplementary Movie 1
Description: A real time movie of the active clusters.
